# Individualized respiratory exercise device originated in Türkiye: design, prototype, and first results

**DOI:** 10.55730/1300-0144.6142

**Published:** 2025-10-26

**Authors:** Çiğdem EMİRZA CİLBİR, Özge ERTAN HARPUTLU, Hikmet UÇGUN, Büşra ÜLKER EKŞİ, Sarper KARA, Esen KIYAN, Gökşen KURAN ASLAN, Buket AKINCI

**Affiliations:** 1 Department of Physiotherapy and Rehabilitation, Faculty of Health Sciences, İstanbul Kültür University, İstanbul, Turkiye; 2 Department of Physiotherapy and Rehabilitation, Institute of Graduate Studies, İstanbul University-Cerrahpaşa, İstanbul, Turkiye; 3 Department of Physiotherapy and Rehabilitation, Faculty of Health Sciences, İstanbul Atlas University, İstanbul, Turkiye; 4 Department of Physiotherapy and Rehabilitation, Institute of Graduate Education, Biruni University, İstanbul, Turkiye; 5 Department of Physiotherapy Program, Vocational School of Therapy and Rehabilitation, İstanbul Galata University, İstanbul, Turkiye; 6 Division of Biomedical Device Technology Program, Vocational School, Biruni University, İstanbul, Turkiye; 7 Department of Chest Diseases, Faculty of İstanbul Medical, İstanbul University, İstanbul, Turkiye; 8 Department of Physiotherapy and Rehabilitation, Faculty of Health Sciences, İstanbul University-Cerrahpaşa, İstanbul, Turkiye; 9 Department of Physiotherapy and Rehabilitation (English), Faculty of Health Sciences, Biruni University, İstanbul, Turkiye

**Keywords:** Respiratory muscle training, exercise, innovation, breathing

## Abstract

**Background/aim:**

Respiratory muscle training (RMT) and breathing exercises are crucial components in cardiopulmonary rehabilitation. RMT includes inspiratory muscle training (IMT) and expiratory muscle training (EMT); however, existing RMT devices typically support only one of these. Incentive spirometers, which also provide visual feedback for breathing exercises, are frequently used. Achieving an RMT program requires multiple devices, which increases both the cost and complexity of the training regimen. This study aimed to design, calibrate, and test a novel individualized respiratory exercise device that integrates both IMT and EMT functions into a single unit, thereby offering a cost-effective, user-friendly alternative for comprehensive cardiopulmonary rehabilitation.

**Materials and methods:**

The design process involved a comparative analysis of existing RMT devices. A concept design was developed, followed by a three-dimensional-printed prototype. Calibration was performed using a pressure meter, with tests conducted on ten healthy volunteers across a range of inspiratory and expiratory pressures. Additionally, a pilot clinical study involving six healthy adults assessed the device’s impact on maximum inspiratory and expiratory pressures (MIP, MEP) and functional exercise capacity during an eight-week RMT period.

**Results:**

The internal consistency of the lowest (0.919) and the highest (0.841) levels of inspiration pressure, and of the lowest (0.901) and the highest (0.856) levels of expiration pressure, were highly reliable. Each healthy participant who completed the eight-week RMT program showed increases in MIP, MEP, and six-minute walking distance.

**Conclusion:**

The novel respiratory exercise device successfully combines IMT and EMT functions with an incentive spirometer, enabling comprehensive respiratory exercises and RMT within a single unit. Its ease of use and adaptability make it a promising option for both clinical and home-based rehabilitation, potentially improving accessibility and adherence to cardiorespiratory rehabilitation protocols. Further clinical studies will investigate the effects of this novel device in cardiopulmonary rehabilitation.

## 1. Introduction

Respiratory exercises and respiratory muscle training (RMT) are crucial components of pulmonary rehabilitation, particularly in patients with chronic respiratory diseases [1,2]. Furthermore, respiratory exercises and RMT are essential components of cardiac rehabilitation, as they improve lung function, enhance oxygen delivery, and strengthen respiratory muscles. These exercises help reduce shortness of breath, increase exercise tolerance, and support overall cardiovascular health, contributing to better outcomes for patients recovering from cardiac events [3,4]. Preoperative RMT reduced the risk of postoperative pulmonary complications and pneumonia while improving maximal inspiratory pressure and shortening hospital stay after cardiac surgery [5]. RMT is also used to increase physical performance and respiratory muscle strength in healthy individuals [6]. These therapeutic interventions have improved the strength and endurance of the respiratory muscles and lung capacity [7].

Various devices, such as incentive spirometers or expiratory muscle training (EMT) devices, are needed for these rehabilitation modalities. Incentive spirometers are respiratory exercise devices commonly used to improve pulmonary ventilation in the early postoperative period during the management of lung diseases, prolonged immobilization, and other conditions requiring improved lung function. Varying mechanical properties facilitate its efficacy [8,9]. Visual feedback provided by these devices, on inspiration or expiration, serves to motivate patients, enhancing their respiratory capacity and adherence to breathing exercises [10]. However, incentive spirometers fail to apply adequate resistance to the respiratory muscles, limiting their effectiveness as tools for RMT [11].

The existing devices, which are used for RMT, primarily provide either inspiratory muscle training (IMT) [12–15] or expiratory muscle training (EMT) [15,16], but rarely both [17,18,19]. This limitation means that patients and healthcare professionals often need to use multiple devices to achieve comprehensive RMT. This not only increases the cost but also complicates the training regimen for both patients and clinicians [8,15].

The high cost of RMT devices and the need for multiple apparatuses present significant barriers to use, especially in resource-constrained settings. This market gap calls for the development of more versatile, cost-effective RMT devices. Such innovations could facilitate rehabilitation, making it more accessible and effective for a broader range of patients. While respiratory exercises and RMT are used in cardiopulmonary rehabilitation, the current devices are limited and often costly. Also, dual-function RMT devices are not common. The primary aim of this study was to present the design and prototype of a novel individualized respiratory exercise device that provides lung ventilation and inspiratory and expiratory muscle training, and to test its calibration by measuring achieved pressures. Also, this study aimed to present preliminary results for participants who have completed RMT using the novel individualized respiratory exercise device.

## 2. Materials and methods

### 2.1. Device design

Incentive spirometer (Voludyne and Triflo), IMT and EMT devices (Threshold IMT, Threshold PEP, Powerbreath), and an example device that can provide both IMT and EMT (PowerLung) were investigated. The working principles and production methods of these devices were examined, cost analyses were performed, and designs were compared. The device models were evaluated in a benchmark test and examined in detail, piece by piece. For each device part, the production method, material, cost, functionality, and quality conditions were determined. The pros and cons, cost, and quality of the devices were determined. An engineer completed the concept design of the novel individualized respiratory exercise device.

### 2.2. Prototype and calibration

**Figure 1:**
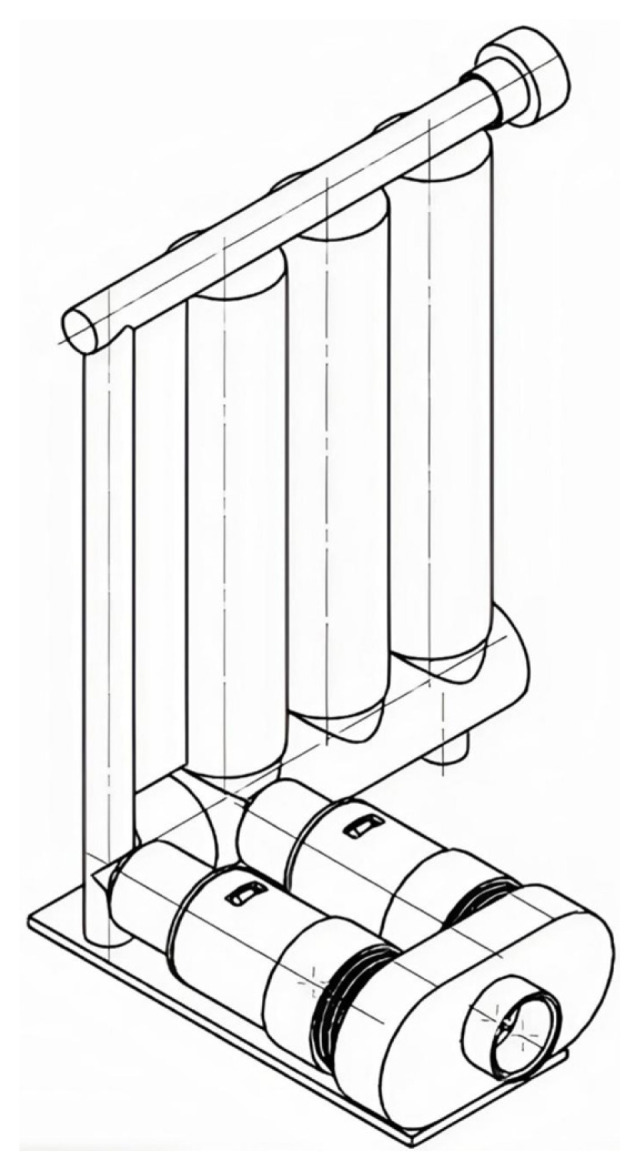
Main technical design of the novel respiratory exercise device.

The main technical design of the novel respiratory exercise device is shown in Figure 1. The prototype of the novel respiratory exercise device comprises several components with specific functions. The spirometer is equipped with a one-way valve that allows air to exit and another that allows air to enter. The colorful balls provide visual feedback to users, indicating the required effort level. To prevent air leakage, the device features airtight gaskets and a sealing bearing that maintains a connection to the hoses. The resistance can be adjusted by rotating a setting, while a platform stabilizes the entire mechanism.

Biomedical calibration was performed using an Extech HD750 Differential Pressure Manometer by a biomedical engineer and two physiotherapists. The respiratory exercise device was tested with three springs with different diameters (0.3 mm, 0.5 mm, 0.7 mm) using a healthy volunteer. Then, the pressures achievable during inspiration and expiration of the device were evaluated using an Extech HD750 with a 0.3-mm-diameter, 45-mm-long spring in ten volunteers (five women, five men). These assessments were completed in the lowest-intensity inspiration, highest-intensity inspiration, lowest-intensity expiration, and highest-intensity expiration stages. This was repeated three times for each participant. The intraclass correlation coefficient (ICC) was calculated to analyze the reliability of the obtained data.

### 2.3. Features of existing devices

**Figure 2:**
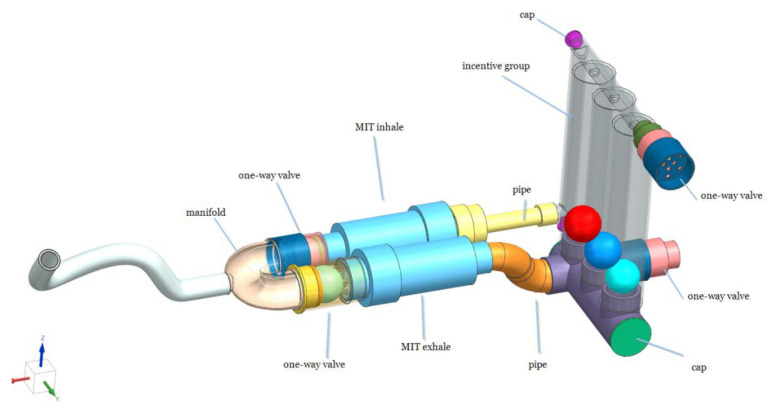
Three-dimensional design of the novel respiratory exercise device.

**Figure 3:**
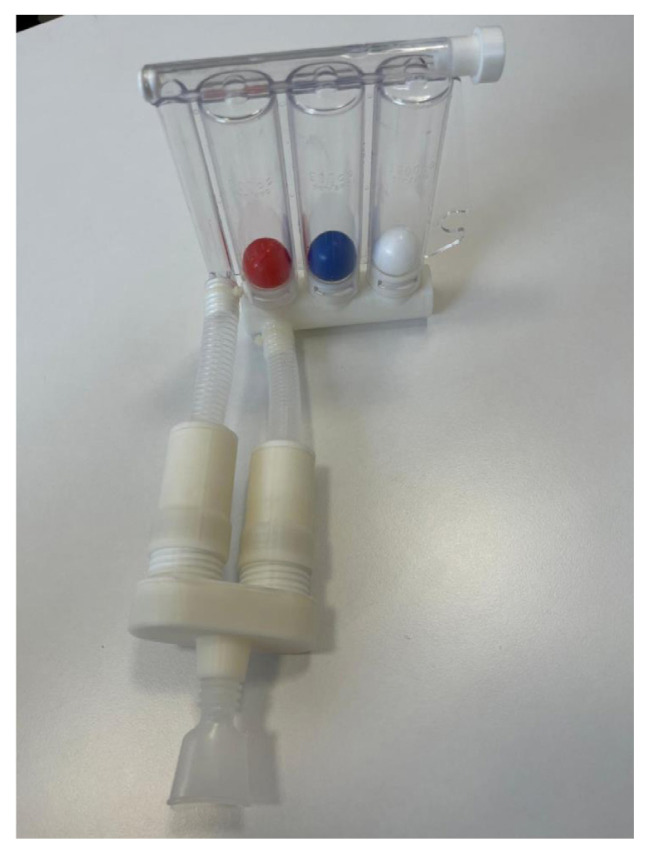
Novel respiratory exercise device.

Following the concept design, a three-dimensional (3D) production design was completed. The first prototype was carried out using a 3D printer (Creality K1) (Figure 2). The parts were then assembled, and the second prototype was developed (Figure 3).

Two different springs are positioned in opposing directions for inspiration and expiration. The valve remains closed during inspiration or expiration until the pressure exceeds the spring resistance, thereby preventing airflow. Once a certain pressure threshold is exceeded, the valve opens, allowing the Triflo component to function. The valve segments are independently designed for airflow in the inspiratory and expiratory directions. The main structure housing the spring is threaded, allowing forward and backward rotation to adjust the spring tension. This adjustability allows the spring to be compressed or released by turning, and different springs can also be used. By keeping the spring length constant and modifying its thickness, thereby altering the spring constant (k), the device’s difficulty level can be adjusted. As a result, individuals can be trained at varying resistance levels as their respiratory muscle strength improves, without the need for an additional device. The formula can represent these levels as follows:


‘F=-k.X’

F indicates the spring’s tension force, k the spring constant, and X the amount of compression or extension. As X increases, the force of the spring also rises. Adjusting the device’s body alters the X value, while changing the spring alters the k value.

The individual using the novel respiratory exercise device initially applies high pressure to try to open the closed valve. Once the valve opens, air reaches the Triflo section. Sustained airflow lifts the balls in the Triflo, with the number of elevated balls increasing as airflow pressure and volume increase. The opening of the valve indicates respiratory muscle strength, and the ball’s movement in the Triflo indicates lung capacity.

### 2.4. Pilot clinical study in healthy individuals

The first clinical phase of this project was approved by the University Clinical Research Ethical Committee (No. 2015-KAEK-682201) and registered at ClinicalTrials.gov (No. NCT06245928).

The pilot study involved six healthy individuals aged 18 or older without any chronic diseases. All participants provided informed consent. Demographic features were collected, and respiratory muscle strength and functional exercise capacity were assessed before participants began the RMT program using the novel respiratory exercise device. Maximum inspiratory pressure (MIP) and maximum expiratory pressure (MEP) were assessed with a spirometer device (COSMED Pony FX; COSMED). The highest value of the three assessments was recorded [20]. The percentages of MIP and MEP values were shown based on reference values [21]. The six-minute walk test (6MWT) was used to determine the functional exercise capacity. The 6MWT was conducted according to the American Thoracic Society/European Respiratory Society guideline and was performed using the Spiropalm 6MWT device (COSMED) [22].

The participants trained with the novel individualized respiratory exercise device for five days per week for eight weeks. Participants were asked to rest following five breathing cycles and repeat a total of 25 breathing cycles (five sets) for both inspiration and expiration in each session. Progression increased by 5–10% weekly, with perceived exertion in the range of 4–6 on the Modified Borg Scale. A chart was also given to participants to record the sessions that they had completed. This chart was checked by the physiotherapist every two weeks.

## 3. Results

### 3.1. Results of calibration

Calibration tests were conducted on 10 healthy volunteers (five women, five men; mean age = 31.5 years; mean body mass index = 24.88 kg/m^2^). Table 1 shows the pressure values obtained at the lowest and highest levels of both inspiration and expiration directions. The internal consistency of the Level 1 (0.919) and Level 5 (0.841) pressures of inspiration, and of the Level 1 (0.901) and Level 5 (0.856) pressures of expiration, was found to be strongly reliable. With the prototype device, a pressure of 21.3–59.5 cmH_2_O was achieved during inspiration with a 0.3 mm-diameter spring. A maximum pressure of 17.2–62 cmH_2_O was achieved for expiration with the same spring.

### 3.2. Comparison with existing devices

Table 2 presents the key technical characteristics of the devices on the market and the novel individualized respiratory training device, aiding professionals in comparing and selecting the most appropriate option based on the patients’ conditions and specific training goals [15].

### 3.3. Classification based on technological innovation system

The Technological Readiness Level (TRL) provides a standardized method to assess the maturity of a technology during its development. This classification facilitates consistent discussions of technical readiness across various technologies. TRL is determined according to program concepts, technology needs, and demonstrated capabilities [23]. The TRL scale ranges from Levels 1–5, with Level 9 representing the most mature technology. This novel device is now at Level 5 of the TRL Production Stage. Level 5 means that “technology validated in a relevant environment” [24].

### 3.4. Case series

Six participants (four women, two men; mean age = 32 years; mean body mass index = 24.66 kg/m^2^) were included in a pilot study and completed an eight-week RMT program using the novel individualized respiratory muscle device. Table 3 presents the baseline and post-training results for MIP, MEP, and the six-minute walking distance (6MWD) of individuals. After eight weeks of training, improvements were observed in each participant’s MIP, MEP, and 6MWT.

## 4. Discussion

This study validated the conceptual design, calibration, and efficacy of a novel respiratory exercise device that combines the clinical features of both an incentive spirometer and RMT devices, offering customization for specific therapeutic goals. The device’s calibration showed an appropriate pressure range for sedentary individuals and those with chronic conditions such as respiratory and cardiac disease. The calibration results were found to be highly reliable. RMT via the novel individualized respiratory exercise device improved MIP, MEP, and functional exercise capacity in healthy individuals. These pilot results indicate that individuals could strengthen the respiratory muscles toward inspiration and/or expiration with wide loading intervals using the same device.

Incentive spirometers provide patients with visual feedback by raising the balls/block during inspiration or expiration, reflecting the individual’s respiratory capacity and aiding in achieving the targeted lung volume. The visual feedback feature is one of the main reasons for its widespread use in physiotherapy and rehabilitation [9,11]. Similarly, our novel individualized respiratory exercise device is designed so that the balls move upwards when used as an incentive spirometer. Unlike the existing RMT devices, our new device provides visual feedback by enabling the balls to move during RMT. With this feature, we anticipate increased adherence to rehabilitation sessions among device users.

Although incentive spirometer is one of the respiratory exercise methods that can increase pulmonary ventilation, it does not provide resistance to the respiratory muscles. The American Thoracic Society and European Respiratory Society pulmonary rehabilitation guidelines highlight the need to use devices that provide resistance to strengthen the respiratory muscles [25,26].

However, most RMT devices only allow inspiratory or expiratory muscle training. So, RMT programs require at least two different devices [15]. Threshold® IMT (Respironics Inc., USA) and Threshold™ PEP (Respironics Inc., USA) devices, which are frequently used in studies and clinical practice, do not offer a sufficient pressure range. The strengths of the devices used for RMT include portability, suitability for home use, and ease of use. Like these devices on the market, the novel individualized respiratory exercise device is easy to use, has easily adjustable features, and can be used in the clinic and at home. Additionally, the strengths of our device include a wider pressure range, support for various breathing exercise programs, and visual feedback.

The wide range of pressure levels is comparable to those of other existing RMT devices [15], and it can be extended by changing the springs to reach higher pressures. There is a limited guide on how the pressure ranges of existing devices are determined. It is questionable whether the variation in difficulty levels of these devices, which offer a range of pressures, is equal. To resolve this confusion, the pressure levels achievable at the hardest and easiest levels of this novel device were tested using a calibration method. Individuals were selected from sex- and age-matched groups to provide objective calibration results. The device’s levels can be adjusted in two ways: first, by changing the level settings, and second, by replacing the spring mechanism within the device.

It was known that RMT improves respiratory muscle strength and exercise performance in healthy individuals [7]. The preliminary results of our study showed that an IMT and EMT program completed using the novel respiratory exercise device improved respiratory muscle strength and functional exercise capacity in healthy people. It is anticipated that providing both IMT and EMT with the same device will be preferred by individuals. There is also a comparator group aiming to investigate the effects of RMT using Threshold® IMT (Respironics Inc, USA) and Thereshold^TM^ PEP (Respironics Inc, USA) devices in this clinical phase of the project. When the required sample size is reached, the results of both groups will be shown to analyze similarities and differences in the effects of RMT via the novel individualized respiratory exercise device and existing devices.

In recent years, RMT has been used not only for cardiopulmonary diseases but also in neurological conditions, chronic kidney disease, and sports performance [27–30]. Recent studies have shown that RMT can improve respiratory muscle strength and functional capacity in these populations [27–30]. These findings suggest that respiratory exercise devices, such as the novel, individualized device used in our study, may become more commonly used in different rehabilitation programs.

There are certain limitations regarding the design and working principle of this device. While it was initially designed to perform IMT and EMT within a single breathing cycle, practical applications have shown that this may not be suitable for all patient populations. Therefore, it is believed that conducting IMT and EMT separately for each phase would be more effective. From a design and functionality perspective, the device falls into the category of resistive loading devices. However, one limitation of these devices is their dependence on airflow for performance. In individuals unable to generate sufficient inspiratory or expiratory flow, there may not be enough force to activate the one-way valves, resulting in suboptimal device performance. Similarly, in patients who cannot achieve adequate airflow, the visual feedback feature provided through the incentive spirometer may not function effectively.

## 5. Conclusion

The novel individualized respiratory exercise device is an easy-to-use, wide-pressure-range device that provides visual feedback. With this new device, three different respiratory exercise programs can be performed on a single device. In the next phase of the project, the device’s clinical effects will be examined in additional groups (healthy individuals, patients with chronic obstructive pulmonary disease, and individuals who have undergone cardiac surgery). Future results will provide more detailed, objective data on its clinical use.

## Figures and Tables

**Table 1:** Intraclass correlation coefficient of the pressures.

	Difficulty	Mean value (min) – (max)	ICC
Inspiration	Level 1	−39.13 (−27.7)–(−54.7)	0.919
	Level 5	−40.07 (−21.3)–(−59.5)	0.841
Expiration	Level 1	38.71 (17.2)–(56.1)	0.901
	Level 5	45.47 (21.3)–(62)	0.856

ICC: intraclass coefficient

**Table 2:** Key features of existing devices and the novel individualized respiratory training device.

Device	Portable	User-friendliness	Proper mouthpiece fit	Suitability for home-based training	Easy to adjust	Allows IMT and EMT
Pflex	Yes	Yes	No	Yes	Yes	Yes
TrainAir	No	No	Yes	No	No	No
POWERbreathe K-Series	Yes	Yes	Yes	Yes	Yes	No
EMST 150	Yes	Yes	No	Yes	Yes	Yes
Orygen-Dual Valve	Yes	Yes	Yes	Yes	Yes	Yes
POWERbreathe	Yes	Yes	Yes	Yes	Yes	Yes
PowerLung	Yes	Yes	Yes	Yes	Yes	No
Respifit-S	Yes	Yes	Yes	No	Yes	No
Threshold IMT	Yes	Yes	No	Yes	Yes	Yes
Threshold PEP	Yes	Yes	No	Yes	Yes	Yes
SpiroTiger	Yes	No	Yes	No	No	No
**Novel individualized respiratory exercise device**	**Yes**	**Yes**	**Yes**	**Yes**	**Yes**	**Yes**

IMT: inspiratory muscle training, EMT: expiratory muscle training

**Table 3:** Baseline and posttraining results of respiratory muscle strength and exercise capacity.

Participant ID	Age	BMI (kg/m^2^)	Baseline MIP (mmHg) (%predicted)	Posttraining MIP (mmHg) (%predicted)	Baseline MEP (mmHg) (%predicted)	Posttraining MEP (mmHg) (%predicted)	Baseline 6MWD (m) (%predicted)	Posttraining 6MWD (m) (%predicted)
1	34	23.50	105.00 (152)	107.00 (153)	127.00 (154)	179.00 (197)	553.00 (84.10)	627.00 (95.40)
2	32	23.50	97.00 (97)	105.00 (112)	79.00 (89)	110.00 (101)	608.00 (87.86)	622.00 (89.88)
3	38	23.40	80.00 (120)	83.00 (123)	92.00 (106)	95.00 (108)	614.00 (85.63)	653.00 (91.07)
4	33	25.70	119.00 (112)	135.00 (127)	130.00 (93)	141.00 (100)	628.00 (82.09)	652.00 (85.13)
5	30	28.80	111.00 (154)	119.00 (165)	75.00 (84)	81.00 (91)	607.00 (101.50)	640.00 (107.90)
6	27	23.10	133.00 (150)	146.00 (163)	134.00 (92)	148.00 (97)	598.00 (69.20)	644.00 (74.50)

BMI: body mass index, MIP: maximum inspiratory pressure, MEP: maximum expiratory pressure, 6MWD: six-minute walking distance
